# A julolidine-fused anthracene derivative: synthesis, photophysical properties, and oxidative dimerization†

**DOI:** 10.1039/c8ra02205d

**Published:** 2018-04-11

**Authors:** Zeming Xia, Xiaoyu Guo, Yanpeng Zhu, Yonggen Wang, Jiaobing Wang

**Affiliations:** School of Chemistry, Sun Yat-Sen University Guangzhou 510275 People's Republic of China wangjb5@mail.sysu.edu.cn

## Abstract

We describe the synthesis and characterization of a julolidine-fused anthracene derivative J-A, which exhibits a maximum absorption of 450 nm and a maximum emission of 518 nm. The fluorescent quantum yield was determined to be 0.55 in toluene. J-A dimerizes in solution *via* oxidative coupling. Structure of the dimer was characterized using single crystal X-ray diffraction.

## Abstract 2

A julolidine fused anthracene derivative with unique photophysical and redox properties was presented.

## Graphical abstract

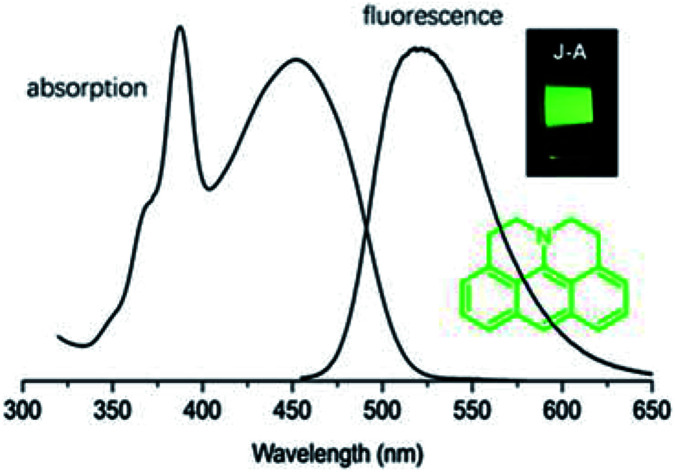

Julolidine^1^ is a popular structural subunit in various fluorescent dyes (Chart 1).^2^ The restricted motion and strong electron-donating capability of the fused julolidine moiety are quite effective for improving the photophysical properties. For instance, julolidine-fused fluorophores normally display desirable photophysical characteristics, such as high quantum yield, red-shifted absorption and emission, and good photostability. Recently, julolidine derivatives have been widely exploited in various applications such as sensing,^3^ imaging,^4^ and nonlinear optical materials.^5^ Several julolidine dyes have been used in dye-sensitized solar cells due to their large π-conjugated system and the promising electron donating property.^6^

**Chart 1:**
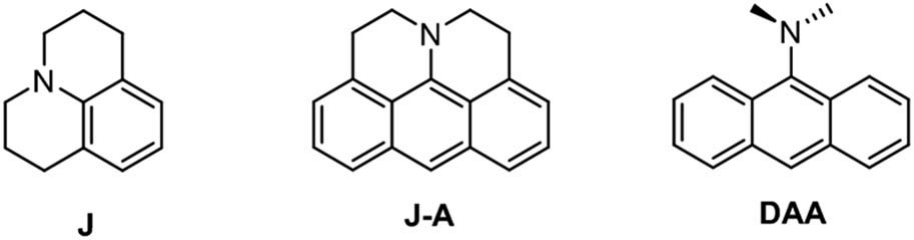
The structure of julolidine, J-A and DAA.

In this paper, we report a julolidine-fused anthracene derivative J-A, which exhibits attractive photophysical properties not observed in DAA, a dimethyl-amino substituted analogue. Both the absorption and emission of J-A show a dramatic red-shift (*ca.* 74 and 131 nm, respectively), compared with the unmodified anthracene (Fig. 2). The fluorescence quantum yield of J-A was determined to be 0.55 in toluene, while the emission of DAA was completely quenched. The observed spectral properties were rationalized by DFT calculations. In addition, we found that J-A was stable in the solid state, but reactive in solution. J-A dimer was formed through oxidative coupling at the para-position of the N-atom in a dichloromethane solution under air atmosphere. The structure of the dimerized product was characterized using single crystal X-ray diffraction, which unambiguously reveals the structural feature of the julolidine-fused anthracene compound. Preparation of J-A is shown in Scheme 1. Detailed synthesis and characterizations are provided in the ESI.†

**Scheme 1:**
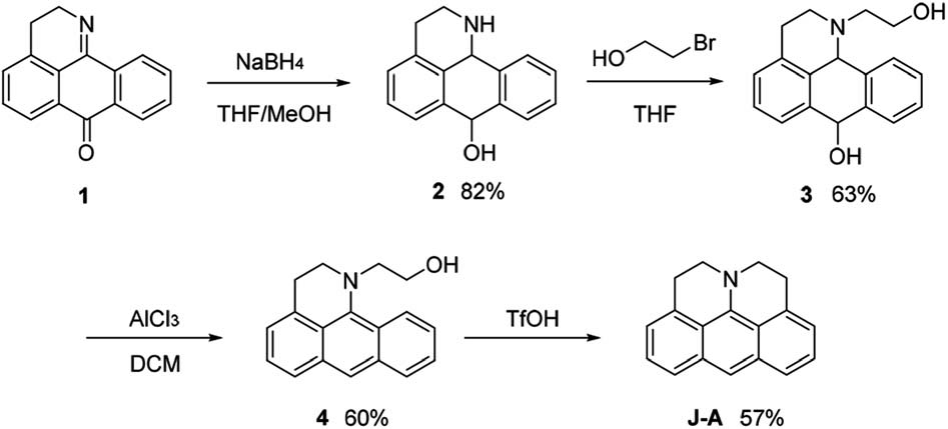
Synthetic route of J-A.

^1^H-NMR signals of J-A shift to the high-field significantly, compared with DAA (Fig. 1), which indicates that the fused structure of J-A facilitates electron delocalization from the nitrogen atom to the anthracene moiety, and thus resulting in a stronger shielding effect. In the case of DAA, however, electron delocalization from the dimethyl amino group to the anthracene core is essentially inhibited due to steric hindrance, which will explain the fact that it displays a spectral feature similar to that of the unmodified anthracene.

**Fig. 1:**
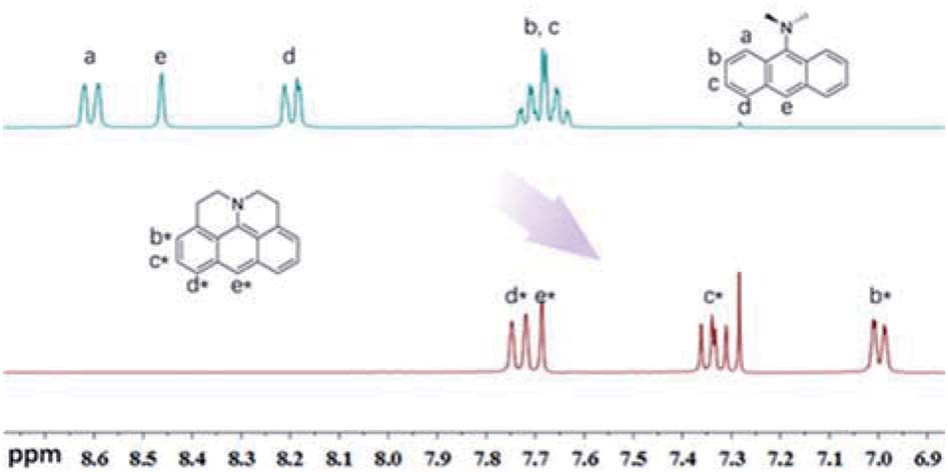
Comparison of the ^1^H-NMR spectrum between J-A and DAA in CDCl_3_. Partial resonance signals in aromatic region are shown.

Fusing with julolidine will exert significant effects on the photophysical properties of anthracene. The absorption and fluorescence spectra of J-A, DAA, and anthracene are shown in Fig. 2. The maximum absorption of J-A is 450 nm, which displays a red-shift of about 70 nm compared with the unmodified anthracene. J-A emits green light (^max^*λ*_em_ = 518 nm, Φ = 0.55), while anthracene emits blue light (^max^*λ*_em_ = 380 nm, Φ = 0.22). In contrast, the absorption of DAA essentially overlaps with that of anthracene, with only a minor red shift of *ca.* 10 nm, but its fluorescence is quenched significantly (Fig. 2). This spectral feature indicates that the dimethyl amino group is electronically separated from the anthracene moiety in the ground state, a result in good accordance with the ^1^H-NMR data shown above. The quenched fluorescence of DAA may result from the photo-induced electron transfer^7^ from the lone pair of the nitrogen atom to the anthracene moiety in the excited state.

**Fig. 2:**
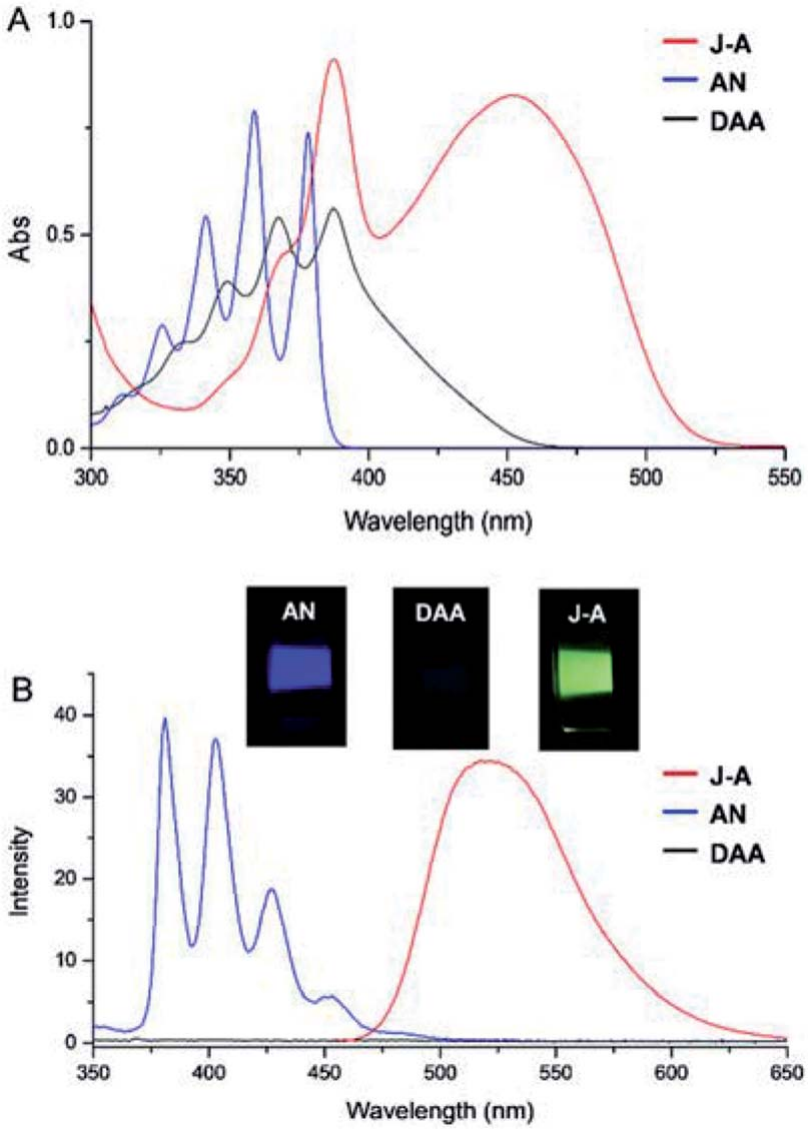
(A) Absorption spectra of J-A, AN, and DAA (1 × 10^−4^ mol L^−1^ in dichloromethane); (B) emission spectra of J-A, AN, and DAA (1 × 10^−5^ mol L^−1^ in dichloromethane). Excitation wavelength: 350 nm. 1 cm cuvette was used in both of the experiments. Inset: visualized fluorescence in solution was shown.

The observed photophysical properties of J-A were reproduced by DFT calculations. The HOMO and LUMO orbitals are evenly distributed over the anthracene moiety and the N-atom in the julolidine, indicating the existence of a conjugated structure. The HOMO–LUMO transition (*f* = 0.10) corresponds to the absorption band at 450 nm. The sharper absorption at 390 nm can be assigned to the HOMO to LUMO + 1 transition. In contrast, the HOMO and LUMO orbitals of DAA resemble those of anthracene, because the dimethyl-amino group is orthogonal to the conjugated π-system (Fig. 3).

**Fig. 3:**
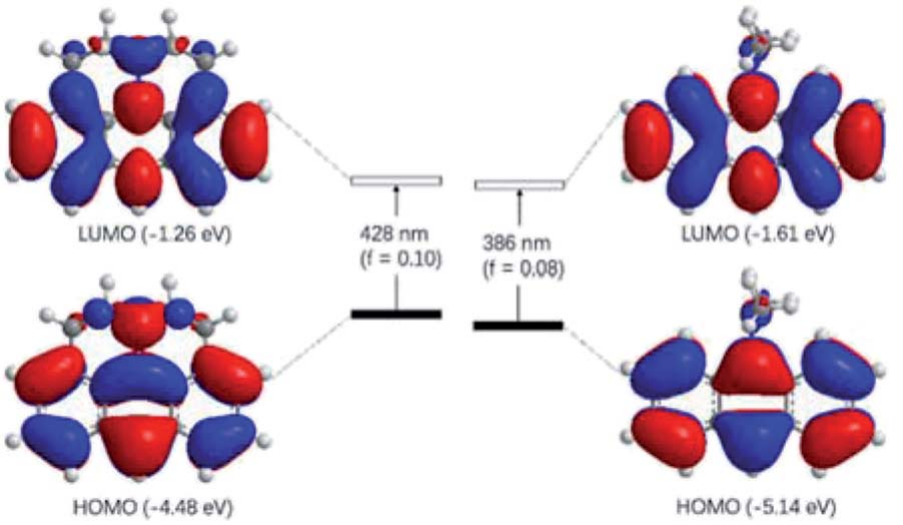
Molecular orbitals of J-A and DAA calculated at the B3LYP/6-31G(d) level of theory (iso value = 0.02). Orbital energies were given in parentheses. Excitation energies were computed by TD-DFT at the same level. Values in parentheses represent the oscillator strengths (f).

J-A is stable in the solid state, but reactive in solution. The cyclic voltammetry (CV) diagram of J-A shows an irreversible oxidation potential at 0.007 V (*vs.* Fc/Fc^+^), indicating that J-A is easy to be oxidized (Fig. S3†). Single crystals suitable for X-ray diffraction study were obtained by slow evaporation of a dichloromethane solution of J-A under air atmosphere. To our surprise, instead of J-A, X-ray data discloses the formation of a dimeric product (Scheme 2) of J-A. We hypothesized that the dimeric compound 5 formed *via* oxidative coupling reaction, a mechanism well-documented for the dimerization of the dimethylaniline compounds.^8^ The ^1^H-NMR spectrum of 5 is distinct from that of J-A. All protons of the anthracene part (b′–d′) appear as a group of multiplet resonance signals (6.93–7.04 ppm) (Scheme 2). In addition, mass spectrometric analysis indicates that two hydrogen atoms were removed after the dimerization of J-A. Compound 5 exhibits a maximum absorption at 460 nm and a very weak emission (^max^*λ*_em_ = 530 nm, Φ = 0.03, Fig. S1†). Two quasi-reversible oxidation waves were identified in the CV diagram of 5 at −0.010 V and 0.135 V (*vs.* Fc/Fc^+^), respectively (Fig. S5†).

**Scheme 2:**
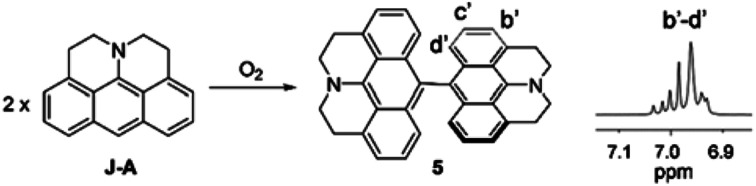
Oxidative dimerization of J-A. Inset: partial ^1^H-NMR of 5 is shown.

X-ray structure of 5 is shown in Fig. 4. The two connected anthracene planes are found to be orthogonal to each other with a dihedral angle of 90.17°, as a result of steric repulsion. Specifically, the bond length of N–C3 is 1.389 Å, which is similar to those of the other reported julolidine compounds (1.359–1.393 Å), while significantly shorter than that of the dimethyl-amino anthracene (1.433 Å).^9^ This result testifies the presence of electron delocalization between the fused julolidine nitrogen and the anthracene π-plane, which is in good agreement with the DFT calculations (Fig. 3). However, the two anthracene π-planes connected by the single bond (C4–C5, 1.489 Å) might not exhibit electron delocalization because of the orthogonal conformation. The fused julolidine ring-i and -ii are symmetric to each other, and both of them adopt an “envelope” conformation. The fused julolidine is nonplanar (bond angle, C3–N–C2, 115.73°, C3–N–C1, 115.92°, C1–N–C2, 113.81°). 5 is closely packed in the crystal (Fig. 4B), and no intercalated solvent molecules were observed. The closest distance between two adjacent anthracene planes is 3.922 Å, indicating a weak π–π stacking. Detailed crystal data are summarized in Table S5.†

**Fig. 4:**
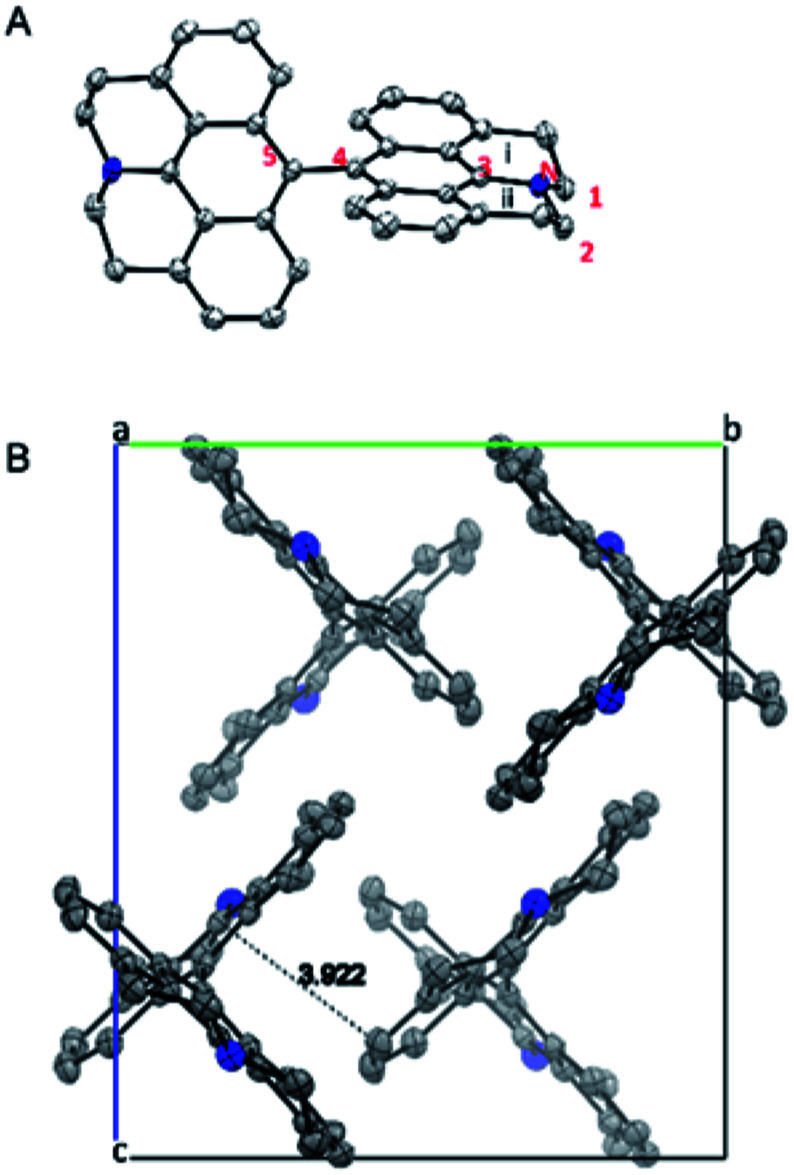
(A) Single crystal X-ray structure of 5. (B) View along *a*-axis.

In summary, we report the synthesis and characterizations of a julolidine-fused anthracene derivative J-A, which demonstrates significantly red-shifted absorption (^max^*λ*_ab_ = 450 nm) and emission (^max^*λ*_em_ = 518 nm, Φ = 0.55), compared with the unmodified anthracene. The photophysical properties of J-A also contrast dramatically with a dimethyl-amino analogue DAA, which were rationalized by DFT calculations. In addition, J-A could be transformed into 5, a dimeric product, whose single crystal X-ray structure unambiguously confirmed the structural feature of the julolidine-fused anthracene.

## Conflicts of interest

There are no conflicts to declare.

## Supplementary Material

RA-008-C8RA02205D-s001

RA-008-C8RA02205D-s002
