# Supplementary material for: A julolidine-fused anthracene derivative: synthesis, photophysical properties, and oxidative dimerization
Source: RSC Adv. 2018 Apr 11;8(24):13588–91. doi: 10.1039/c8ra02205d (PMC9079783; doi:10.1039/c8ra02205d)
Supplement: RA-008-C8RA02205D-s001 [file RA-008-C8RA02205D-s001.pdf]

Electronic Supplementary Material (ESI) for RSC Advances.
This journal is © The Royal Society of Chemistry 2018

Supporting Information

A Julolidine-Fused Anthracene Derivative: Synthesis, Photophysical Properties, and Oxidative Dimerization

Zeming Xia, Xiaoyu Guo, Yanpeng Zhu, Yonggen Wang, Jiaobing Wang*

School of Chemistry, Sun Yat-Sen University, Guangzhou 510275, People's Republic of China.

e-mail: wangjb5@mail.sysu.edu.cn

General remarks

Chemicals were purchased from Energy Chemical, Aldrich, J&K Chemical, and were used without further purification. Freshly distilled tetrahydrofuran (THF), dichloromethane (DCM) were used for synthesis. Compound **1** was synthesized according to a reported procedure,[1a] and the synthesis of compound **4** is based on another literature[1b]. Qingdao Haiyang silica gel (200-300 mesh) was used in flash chromatography. Technical grade solvents were used for extraction and chromatography. NMR spectra were recorded on a Bruker Avance 300 spectrometer (300 MHz). UV-vis absorbance spectra were recorded on a shimadzu UV-2600 spectrophotometer. The emission spectra in solution were recorded on a shimadzu RF-5301PC spectrophotometer. Cyclic voltammetry experiments were carried out at room temperature in argon-purged solutions with CHI660E electrochemical workstation. High-resolution mass spectra were recorded with a Bruker ESI-Q-TOF MaXis 4G mass spectrometer. The single crystal X-ray diffraction data was collected on an Agilent Sapphire3 Gemini Ultra single crystal diffractometer using CuKα radiation.

Contents:

Synthesis……………………………………………S2

Photophysical properties……………………………………………S4

DFT calculations……………………………………………S5

Cyclic voltammetry……………………………………………S7

X-ray crystallographic analysis……………………………………………S8

^{1}H-NMR and ^{13}C-NMR spectra……………………………………………S10

Synthesis

Scheme S1. Synthesis of **J-A**

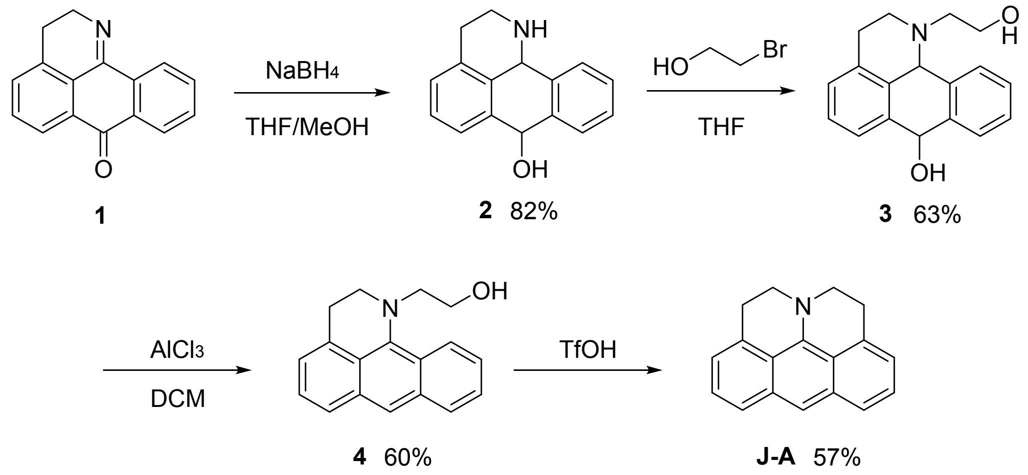

Compound 2: $NaBH_4$ (8.2 g, 210 mmol) was added portionwise to a solution of **compound 1** (5.0 g, 21 mmol) dissolved in a mixed solvent of MeOH (100 mL) and THF (100 mL) under 0 °C. The solution was stirred at 0 °C for 10 min and quenched with H_2O. The reaction mixture was extracted with DCM (50 mL×3), and the organic phase was dried over anhydrous Na_2SO_4 and concentrated under reduced pressure. The residue was purified via silica gel column chromatography with EA/MeOH = 20/1 (V/V) as an eluent, providing **compound 2** as a white solid (4.1 g, yield 82 %). ^{1}H NMR (300 MHz, MeOD) δ 7.77 (dd, J = 5.2, 2.9 Hz, 1H), 7.58 (t, J = 7.0 Hz, 2H), 7.41 – 7.27 (m, 2H), 7.23 (t, J = 7.6 Hz, 1H), 7.05 (d, J = 7.5 Hz, 1H), 5.48 – 5.33 (m, 1H), 4.62 (s, 1H), 3.56 – 3.41 (m, 1H), 3.21 (td, J = 12.0, 3.8 Hz, 1H), 3.03 (ddd, J = 19.2, 15.2, 6.5 Hz, 1H), 2.81 (d, J = 16.8 Hz, 1H). ^{13}C NMR (75 MHz, MeOD) δ 139.87, 139.39, 136.30, 133.10, 132.20, 126.85, 126.17, 126.09, 125.89, 122.45, 122.16, 120.33, 67.25, 53.35, 42.68, 28.12. HRMS (ESI): calcd. for C16H15NO [M+H] +: 238.122$_6$, found: 238.1223.

Compound 3: Compound 2 (2.0 g, 8.4 mmol), K_2CO_3 (5.8 g, 42 mmol) and 2-Bromoethanol (5.3 mg, 42 mmol) were suspended in THF (100 mL). The solution was stirred at 70 °C under N_2 for 12 h. After cooling to room temperature, the reaction mixture was washed with water and extracted with DCM (50 mL×3), the organic phase was dried over Na_2SO_4 and evaporated under reduced pressure. The residue was purified by silica gel column chromatography with DCM as an eluent to provide **compound 3** as a pale yellow oil (1.5 g, yield 63 %).^{1}H NMR (300 MHz, MeOD) δ 7.78 (dd, J = 5.0, 3.7 Hz, 1H), 7.69 (dd, J = 4.6, 3.7 Hz, 1H), 7.51 (d, J = 7.6 Hz, 1H), 7.32 – 7.21 (m, 2H), 7.17 (t, J = 7.6 Hz, 1H), 7.00 (d, J = 7.5 Hz, 1H), 5.44 (s, 1H), 4.22 (s, 1H), 3.89 (ddd, J = 17.2, 11.3, 5.1 Hz, 2H), 3.36 – 3.30 (m, 1H), 3.26 – 3.08 (m, 1H), 2.86 (d, J = 6.5 Hz, 3H), 2.65 (dt, J = 12.9, 5.8 Hz, 1H). ^{13}C NMR (75 MHz, MeOD) δ 141.06, 140.68, 137.50, 133.11, 132.40, 125.91, 125.73, 125.49, 125.26, 123.29, 122.11, 120.04, 67.77, 60.71, 59.55, 26.11. HRMS (ESI): calcd. for C18H19NO2 [M+H] +:282.148$_8$, found: 282.1480.

Compound 4: $AlCl_3$ (2.4 g, 18 mmol) was added portionwise to a solution of **Compound 3** (1.0 g, 3.6 mmol) dissolved in dry DCM (50 mL) under 0 °C. The solution was stirred at 25 °C for 30 min and quenched with H_2O. The reaction mixture was extracted with DCM (50 mL×3), and the organic phase was dried over anhydrous Na_2SO_4 and concentrated under reduced pressure. The residue was purified via silica gel column chromatography with DCM/PE = 1/1 (V/V) as an eluent, providing **compound 4** as a yellow solid (0.7 g, yield 60 %).^{1}H NMR (300 MHz, $CDCl_3$) δ 8.65 – 8.52 (m, 1H), 8.17 (s, 1H), 8.05 – 7.94 (m, 1H), 7.87 (d, *J* = 8.5 Hz, 1H), 7.58 – 7.44 (m, 2H), 7.40 (dd, *J* = 8.5, 6.7 Hz, 1H), 7.18 (dd, *J* = 6.6, 1.0 Hz, 1H), 4.08 (t, *J* = 5.8 Hz, 2H), 3.71 – 3.54 (m, 2H), 3.42 (t, *J* = 5.8 Hz, 2H), 3.27 – 3.14 (m, 2H). ^{13}C NMR (75 MHz, $CDCl_3$) δ 143.04, 132.86, 132.51, 131.63, 128.87, 126.74, 125.32, 124.85, 124.81, 124.66, 123.83, 123.24, 122.45, 121.79, 60.08, 56.03, 44.92, 24.45. HRMS (ESI): calcd. for C18H17NO [M] +:263.130$_4$, found: 263.1331

Compound J-A: Compound 4 (0.5 g, 1.9 mmol) was dissolved in trifluoromethanesulfonic acid (10 mL) and the solution was stirred at 130 °C under N_2 for 12 h. After cooling to room temperature, the reaction mixture was poured into ice water and neutralized with $NH_3{\cdot}H_2O$ (20 mL). The mixture was extracted with DCM (50 mL×3), and the organic phase was dried over anhydrous Na_2SO_4 and concentrated under reduced pressure. The residue was purified via silica gel column chromatography with DCM/PE = 1/3 (V/V) as an eluent, providing **compound J-A** as a yellow solid (0.3 g, yield 57 %).^{1}H NMR (300 MHz, $CDCl_3$) δ 7.73 (d, *J* = 8.5 Hz, 2H), 7.69 (s, 1H), 7.34 (dd, *J* = 8.6, 6.7 Hz, 2H), 7.00 (dd, *J* = 6.6, 1.1 Hz, 2H), 3.49 (dd, *J* = 8.9, 3.5 Hz, 4H), 3.43 – 3.30 (m, 4H). ^{13}C NMR (75 MHz, $CDCl_3$) δ 141.83, 132.91, 132.37, 125.83, 125.45, 119.25, 116.19, 113.16, 50.68, 30.15. HRMS (ESI): calcd. for C18H15N [M] +:245.119$_9$, found: 245.1124.

Compound 5: ^{1}H NMR (300 MHz, $CDCl_3$) δ 7.90 – 7.80 (m, 2H), 7.72 (ddd, *J* = 5.6, 3.1, 1.0 Hz, 2H), 7.36 – 7.20 (m, 5H), 4.11 – 3.84 (m, 2H), 3.02 (dd, *J* = 8.6, 6.8 Hz, 2H). ^{13}C NMR (75 MHz, $CDCl_3$) δ 132.77, 132.46, 125.45, 125.21, 120.84, 119.51, 116.33, 51.04, 30.65. HRMS (ESI): calcd. for C36H28N2 [M+H] +:489.232$_5$, found: 489.2338.

Photophysical properties of J-A and compound 5

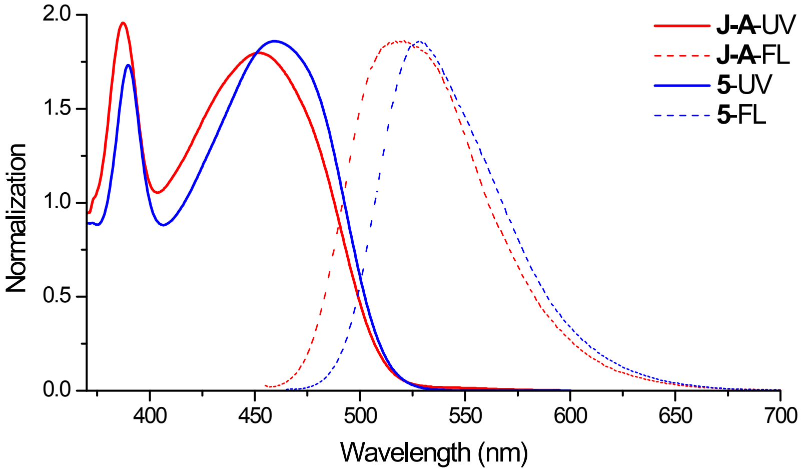

Figure S1 Absorption and emission spectra of **J-A** and **5** (Absorption spectra were recorded at the concentration of 10^{-4} mol/L in dichloromethane in a 1 cm cuvette. Emission spectra were recorded at the concentration of 10^{-5} mol/L in dichloromethane in a 1 cm cuvette. Excitation wavelength: 450 nm.)

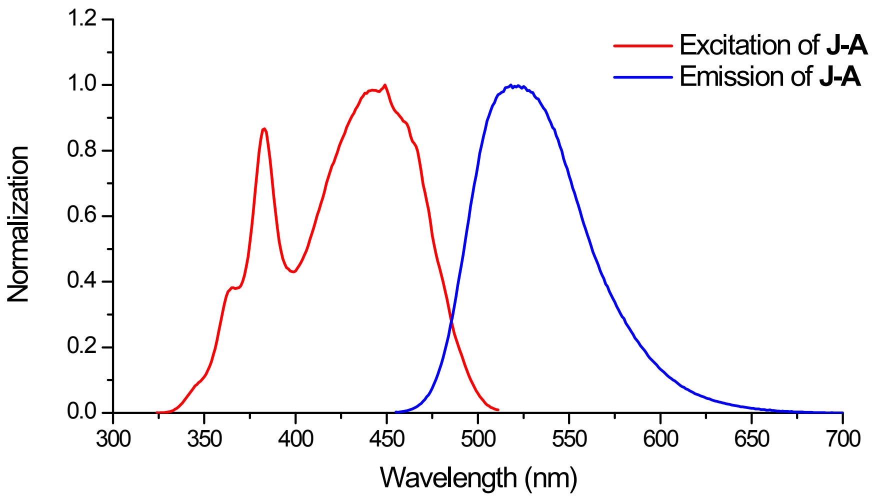

Figure S2 Excitation and emission spectra of **J-A** (the excitation and emission spectrum was recorded at the concentration of 10^{-5} mol/L in dichloromethane in a 1 cm cuvette. The emission wavelength of the excitation spectrum is 520 nm and the excitation wavelength of the emission spectrum is 450 nm.)

Table S1. Quantum yield of compound **J-A** , **5**, **DAA** and **Anthracene**

**Compound**	**J-A**	**5**	**DAA**	**Anthracene**
Φ Quantum yield[a]	0.55	0.02	0.03	0.22

[a]The quantum yields of the compounds were measured in toluene. 9,10-diphenyl-anthracene **DPA** (φ=0.70 in toluene) is used as reference.[2]

DFT Calculations [3]

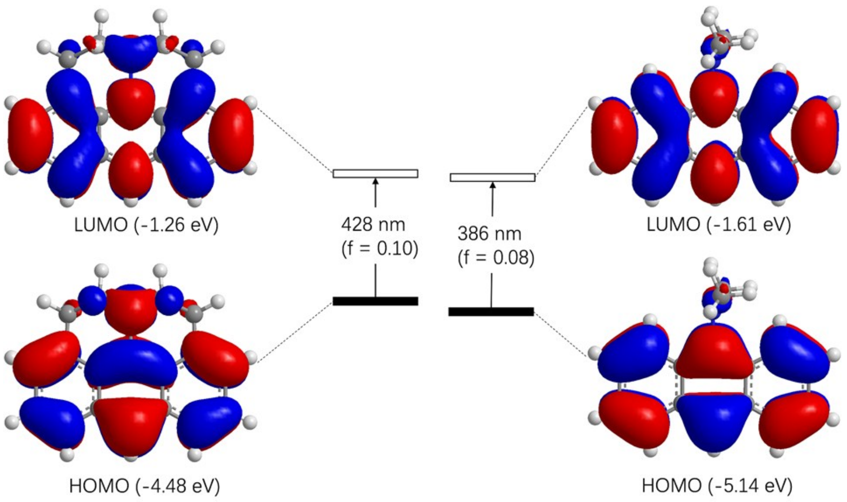

Figure S3. Kohm-Sham frontier orbitals of **J-A** and **DAA** calculated at the B3LYP/6-31G(d) level of theory (isovalue = 0.02). Orbital energies were given in parentheses. Excitation energies were computed by TD-DFT at the same level. Values in parentheses represent the oscillator strengths (f)

Table S2. Excitation energies and oscillator strengths

**Compound**	Transition	Wavelength (nm)	Energy (eV)	Oscillator strength
**J-A**	S_0-S_1	428.44	2.8938	0.0995
	S_0-S_2	337.07	3.6783	0.0546
	S_0-S_3	297.42	4.1686	0.0016
	S_0-S_4	277.90	4.4614	0.0018
	S_0-S_5	263.37	4.7076	0.0000

Table S3. Coefficient of orbital

**Compound**	Transition	Coefficient of orbital	
**J-A**	S_0-S_1	HOMO -> LUMO (0.70165)	
	S_0-S_2	HOMO-1 -> LUMO (-0.35588)	HOMO -> LUMO+1 (0.60759)
	S_0-S_3	HOMO-3 -> LUMO (0.17289)	HOMO -> LUMO+2 (0.67843)
	S_0-S_4	HOMO-2 -> LUMO (0.69566)	
	S_0-S_5	HOMO-3 -> LUMO (0.66983)	HOMO -> LUMO+2 (-0.16807)

Table S4. Cartesian coordinates of optimized structure of **J-A**

H	4.60777871	0.321903	-0.18481
C	3.6604325	-0.20729	-0.11117
C	2.48224431	0.499556	-0.13552
C	2.47204315	-2.3112	0.072689
C	1.22925688	-0.18686	-0.03388
C	3.6552554	-1.6247	-0.00085
C	1.21751825	-1.62576	0.05037
C	0.00000003	0.526474	-0.03592
H	4.60048257	-2.16104	0.018407
H	-0.0000001	-3.39709	0.186273
H	2.46460875	-3.39615	0.144213
C	-1.229257	-0.18686	-0.03388
C	-2.48224434	0.499557	-0.13552
C	-1.21751859	-1.62576	0.05037
H	-2.46461002	-3.39615	0.144213
C	-0.00000019	-2.31253	0.109072
C	-3.66043269	-0.20729	-0.11117
H	-4.60777882	0.321904	-0.18481
C	-3.65525584	-1.62469	-0.00085
H	-4.60048302	-2.16104	0.018407
C	-2.47204379	-2.3112	0.072689
N	0.00000016	1.923249	-0.04243
C	-1.22735073	2.58242	0.3999
H	-1.35457365	2.475488	1.49249

H	-1.13210403	3.65164	0.18217
C	1.22735186	2.582421	0.3999
H	1.35457404	2.475488	1.49249
H	1.13210498	3.65164	0.182169
C	2.43756758	1.995757	-0.32058
H	3.35680262	2.464781	0.048379
H	2.35926263	2.237132	-1.39068
C	-2.43756709	1.995758	-0.32058
H	-2.35926224	2.237133	-1.39068
H	-3.3568017	2.464782	0.048379

Cyclic voltammetry

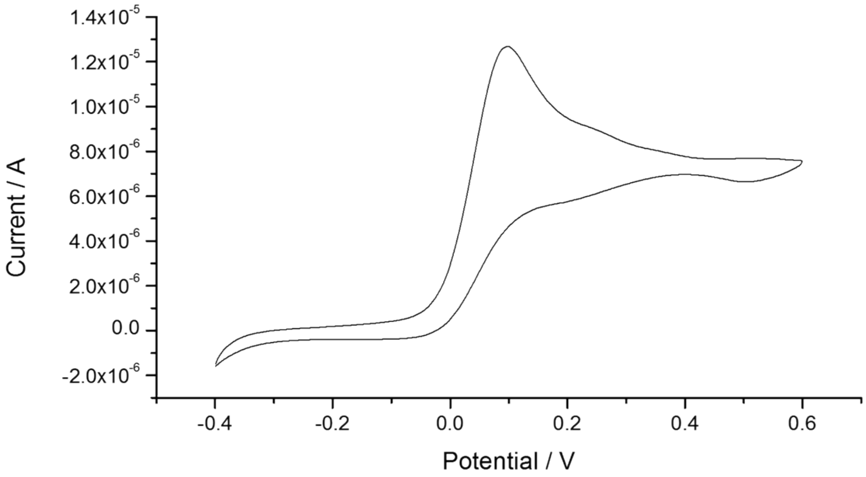

Figure S4. CV diagrams of **J-A** (vs Fc/Fc^{+}) in dichloromethane. One irreversible oxidation wave is identified at 0.007 V. Supporting electrolyte: 0.1 M n-Bu_4NPF_6. Working electrode: Pt disk. Counter electrode: Pt wire. Reference electrode: Ag/Ag^{+}. Scan rate: 10 mV/s.

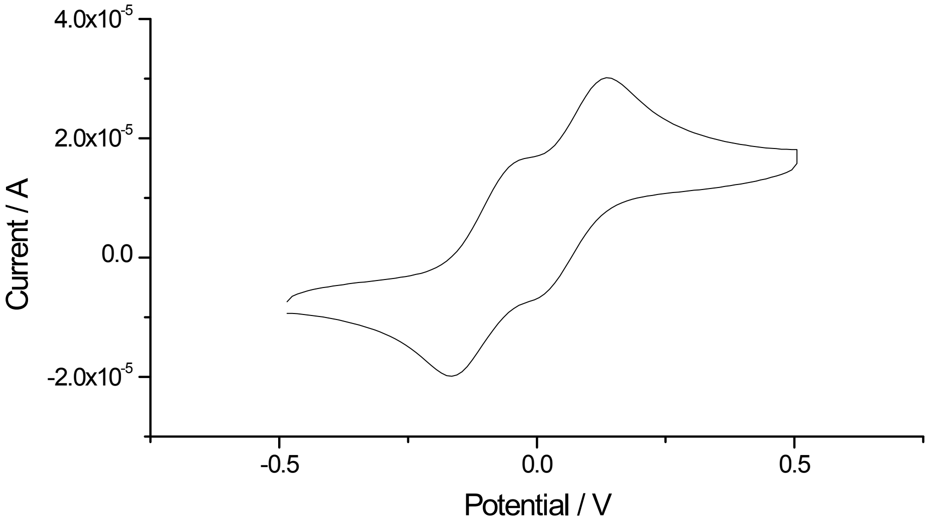

Figure S5. CV diagrams of **5** (vs Fc/Fc$^+$) in dichloromethane. Two oxidation waves are identified at -0.010 V and 0.135 V, respectively. Supporting electrolyte: 0.1 M n-Bu_4NPF_6. Working electrode: Pt disk. Counter electrode: Pt wire. Reference electrode: Ag/Ag$^+$. Scan rate: 500 mV/s.

X-ray crystallographic analysis

Method of crystal growth: 2 mg of **J-A** was dissolved in dichloromethane (1 ml) under air atmosphere. After 12 h, yellow crystals suitable for X-ray structural determination were obtained.

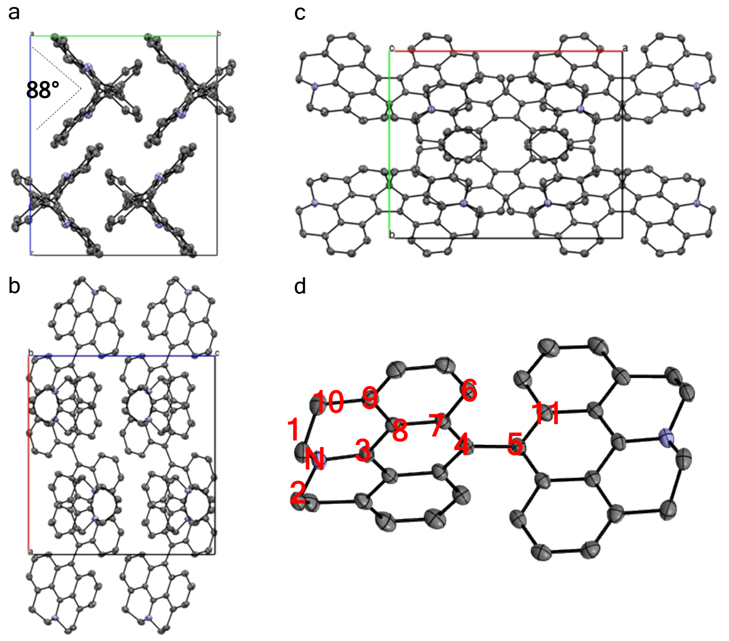

Selected parameters

N- C1	1.458 Å
N- C2	1.464 Å
N- C3	1.390 Å
C1- C10	1.515 Å
C9- C10	1.507 Å
C8- C9	1.431 Å
C3- C8	1.421 Å
C8- C7	1.432 Å
C7- C4	1.408 Å
C7- C6	1.421 Å
C4- C5	1.489 Å
C1- N- C2	113.81°
C1- N- C3	115.92°
C2- N- C3	115.73°
C7- C4- C5- C11	90.17 °

Figure S6. Packing mode of **J-A** in the crystal. View along (a) a-axis; (b) b-axis; (c) c-axis; and (d) X-ray crystal structure of **J-A**, Hydrogen atoms were ommited for clarity. Inset: Representative crystal data are shown.

Table S5. Crystallographic data and refinement details of **5**

Compound	**5**
Formula	C36 H28 N2
MW	488.60
Lattice type	orthorhombic
Space group	PBCN
a /Å	14.7747(3)
b/Å	11.8462(3)
c/Å	13.9054(3)
α/°	90
β/°	90
γ/°	90
V/Å^3	2433.78(9)
Z value	4
ρ_{calc} (g/cm^{-3})	1.334
T /K	150
GOF on F^2	1.032
R_1 [F^2>2σ(F^2)]	0.0409(2056)
wR_2 (all data)	0.1181(2370)
CCDC	1829089

NMR Spectra

^{1}H NMR spectrum of **2**

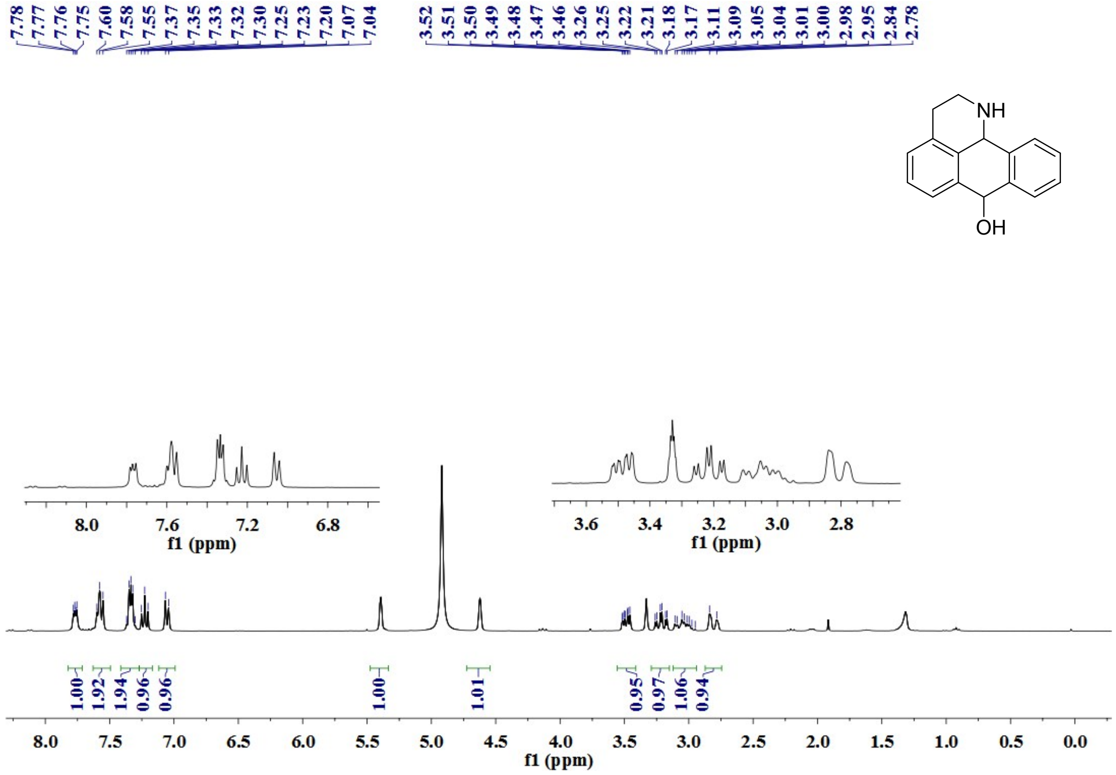

^{13}C NMR spectrum of **2**

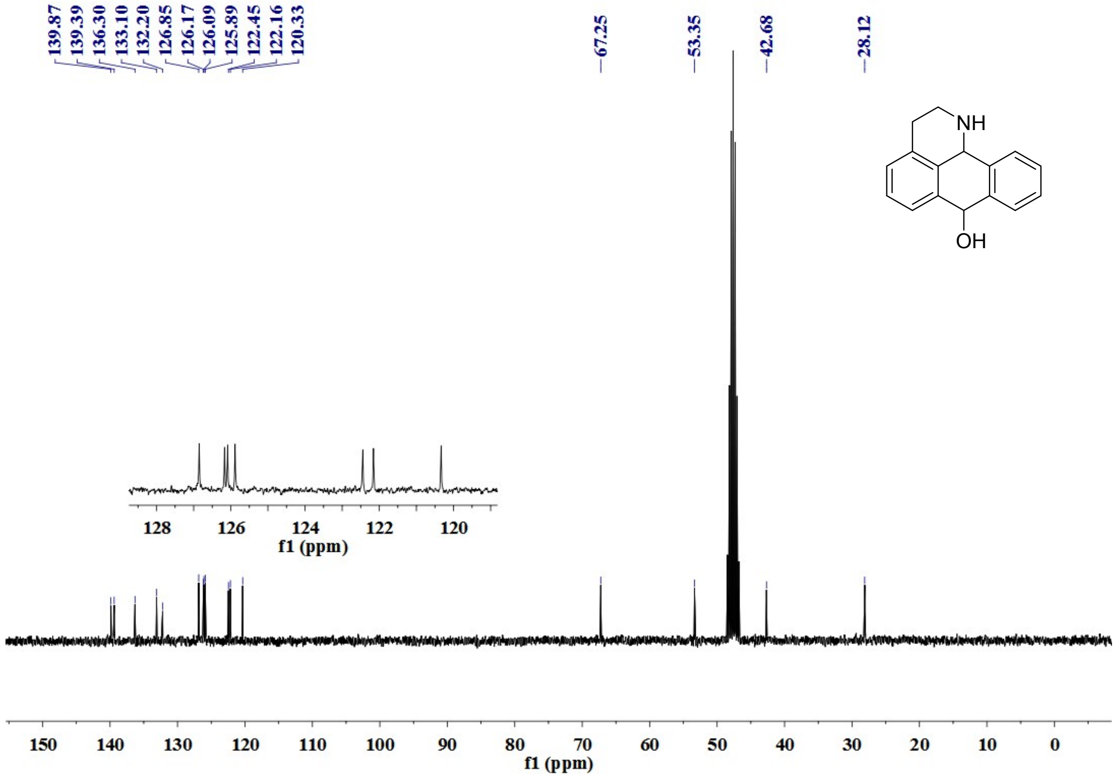

^{1}H NMR spectrum of **3**

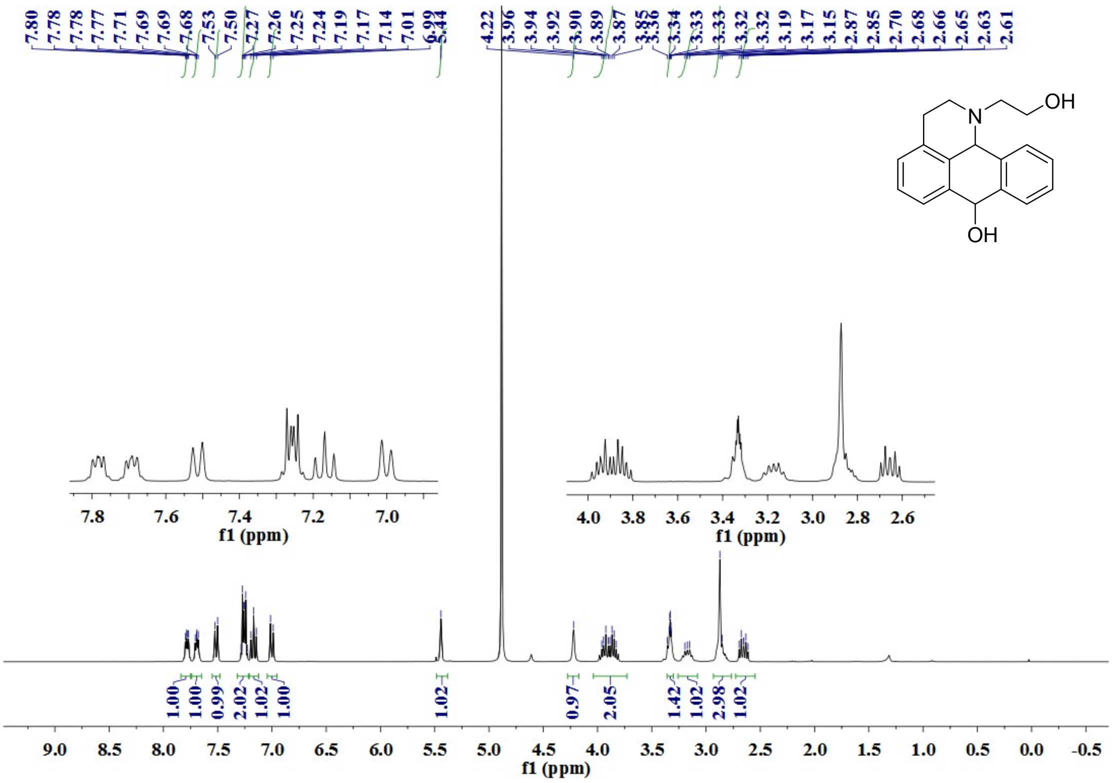

^{13}C NMR spectrum of **3**

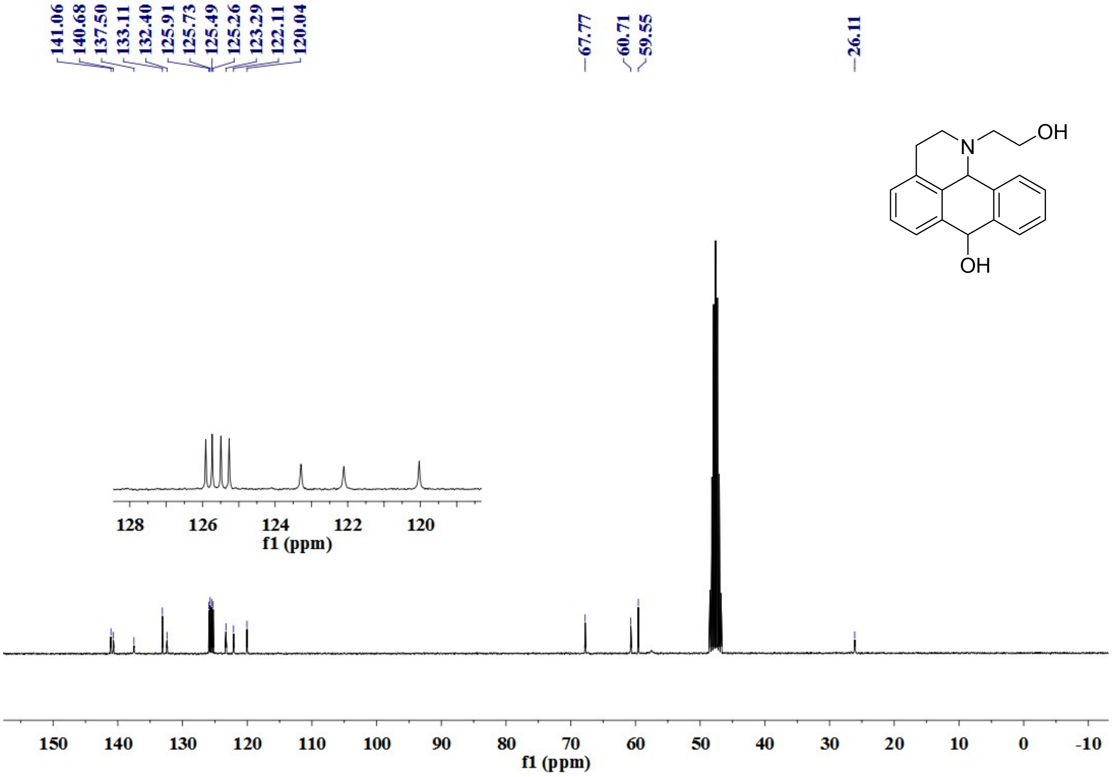

^{1}H NMR spectrum of **4**

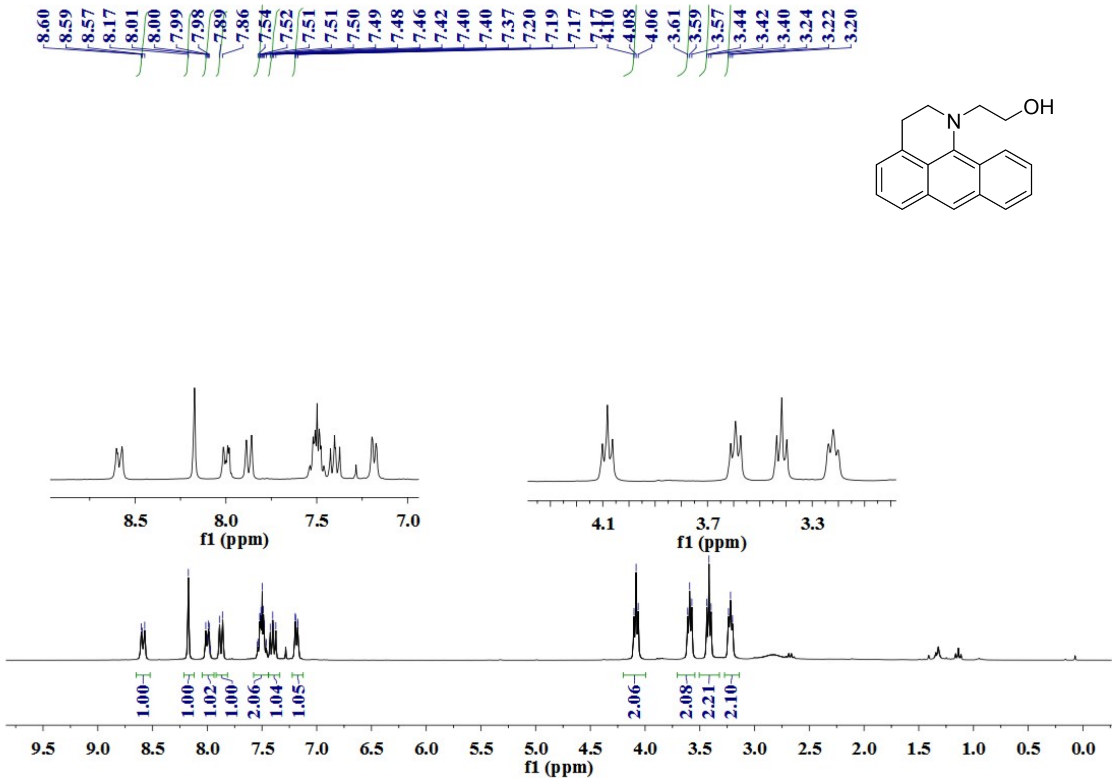

^{13}C NMR spectrum of **4**

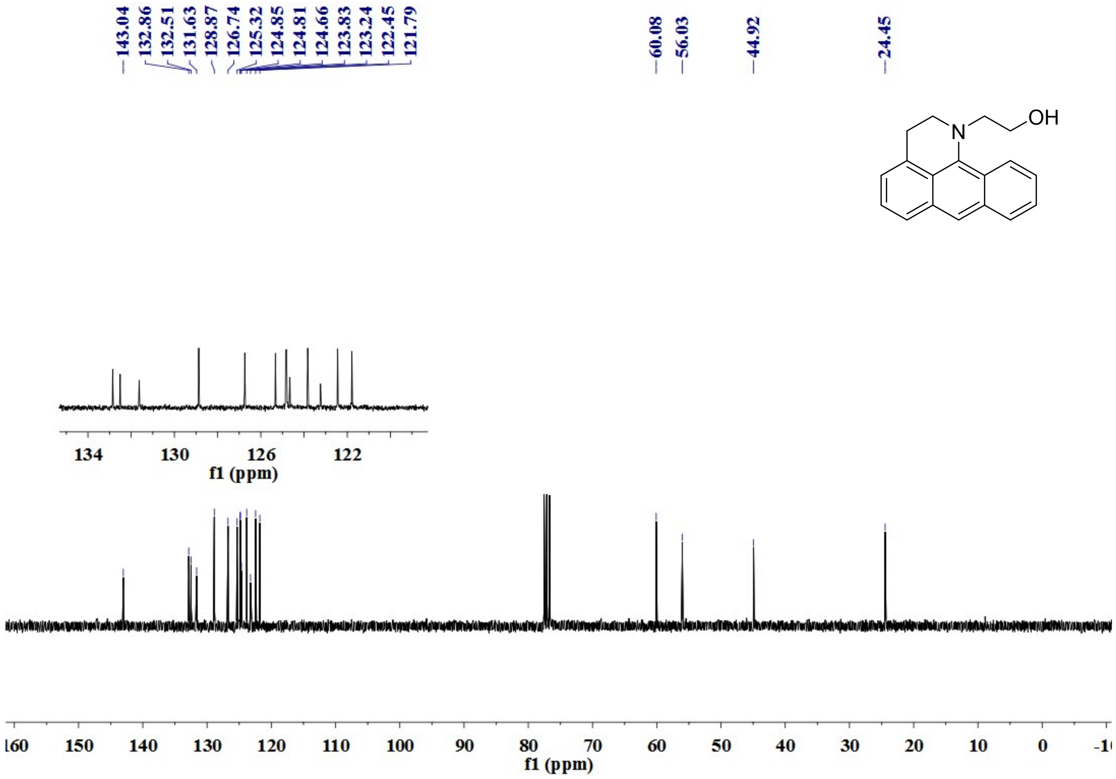

^{1}H NMR spectrum of **J-A**

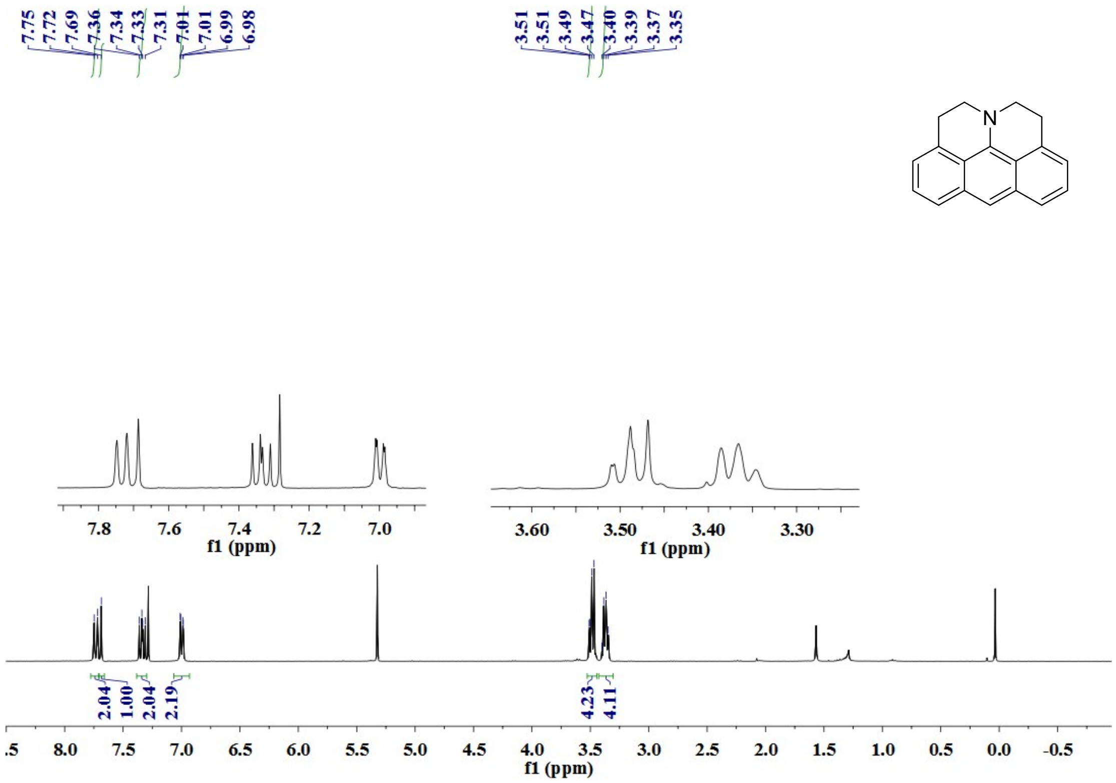

^{13}C NMR spectrum of **J-A**

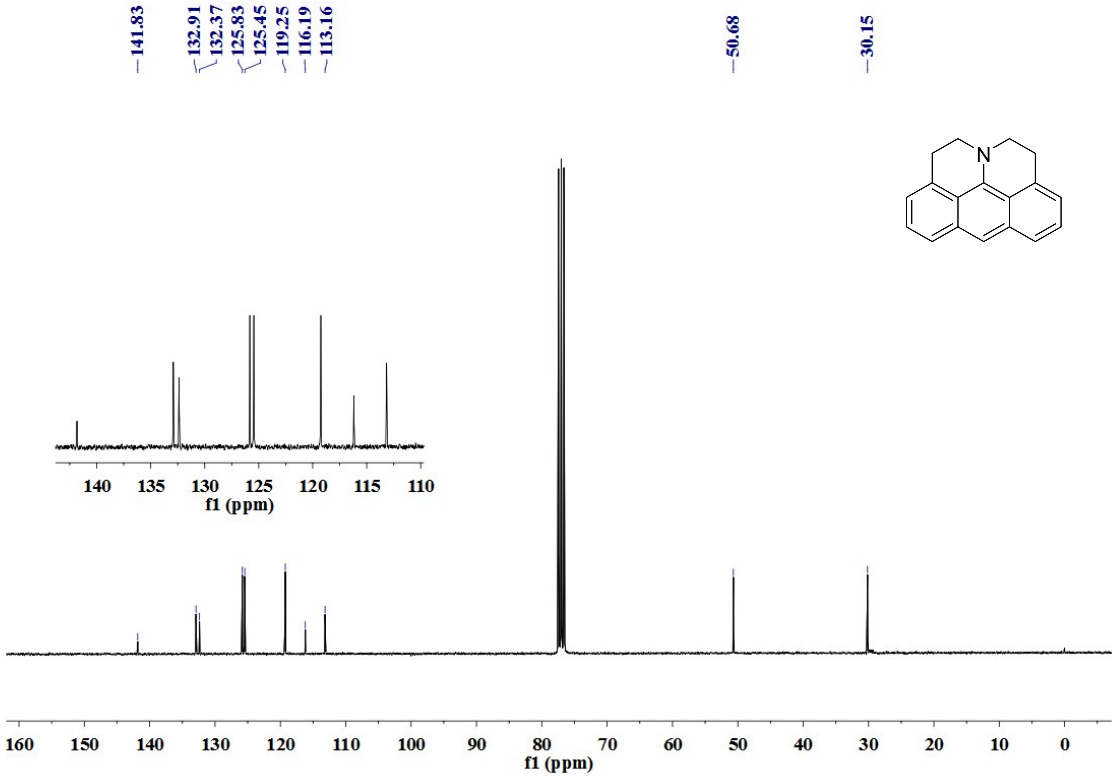

^{1}H NMR spectrum of **5**

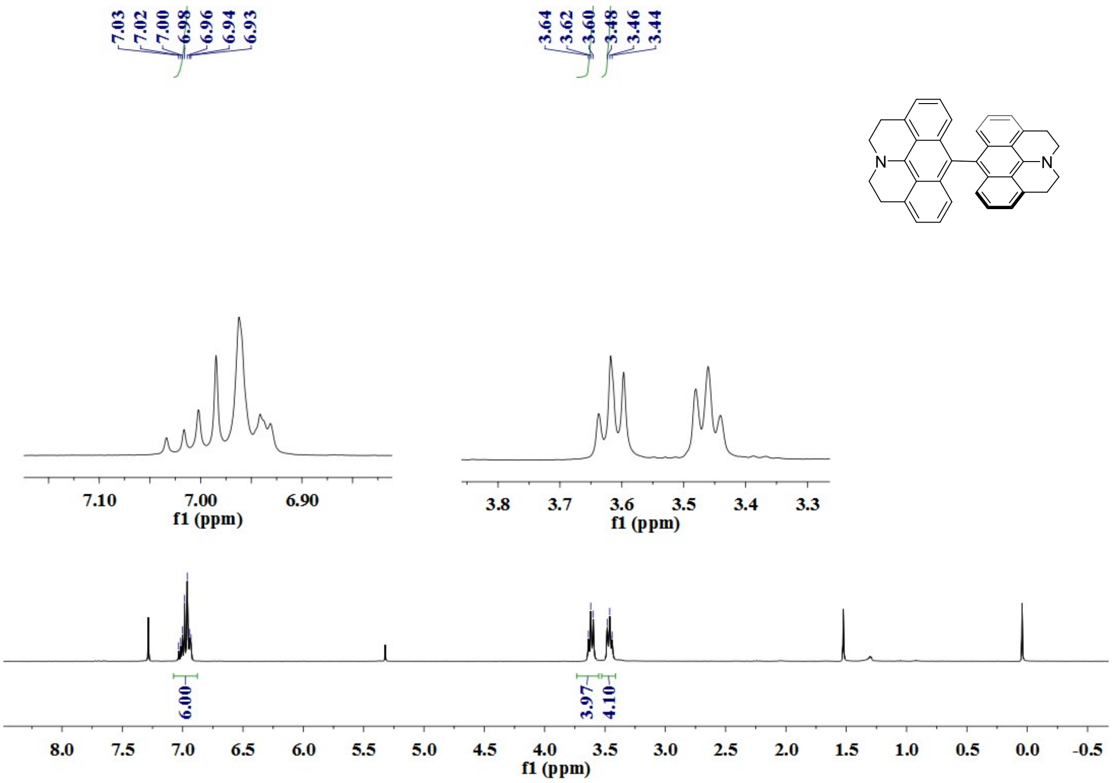

^{13}C NMR spectrum of **5**

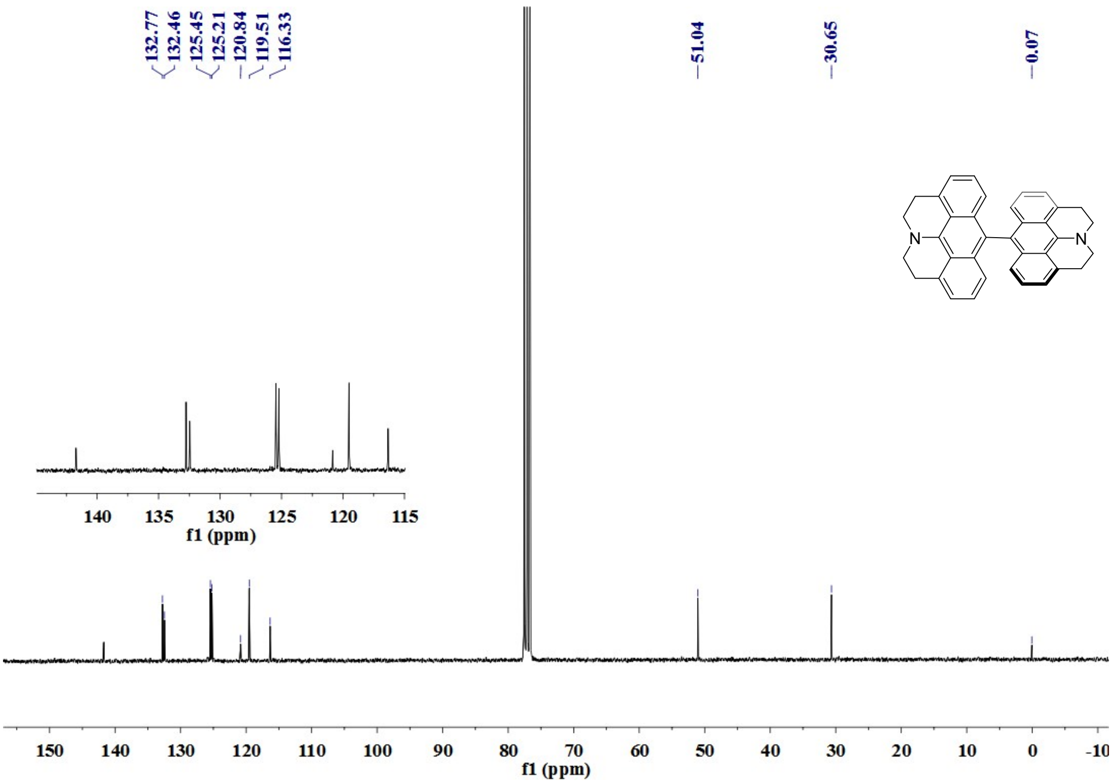

Reference

(1) (a) V. Castro-Castillo, C. Suarez-Rozas, A. Pabon, E. G. Perez, B. K. Cassels and S. Blair, *Bioorg. Med. Chem. Lett.*, 2013, **23**, 327-329. (b) Bremner, J. B.; Jaturonrusmee, W.; Engelhardt, L. M.; White, A. H. *Tetrahedron Lett.* 1989**, 30**, 3213.

(2) Galanin M. D.; Kutenkov A. A.; Smorchkov V. N.; Timofeev Y. P.; Chizhikov Z. A. *Opt. Spektrosk*., 1982, **53**, 683.

(3) Both the geometry optimization and energy calculation were performed by employing density functional theory (DFT), at the level of B3LYP/6-31G(d) on the Gaussian09 program package, M. J. Frisch, G. W. Trucks, H. B. Schlegel, G. E. Scuseria, M. A. Robb, J. R. Cheeseman, G. Scalmani, V. Barone, B. Mennucci, G. A. Petersson, H. Nakatsuji, M. Caricato, X. Li, H. P. Hratchian, A. F. Izmaylov, J. Bloino, G. Zheng, J. L. Sonnenberg, M. Hada, M. Ehara, K. Toyota, R. Fukuda, J. Hasegawa, M. Ishida, T. Nakajima, Y. Honda, O. Kitao, H. Nakai, T. Vreven, J. A. Montgomery, Jr., J. E. Peralta, F. Ogliaro, M. Bearpark, J. J. Heyd, E. Brothers, K. N. Kudin, V. N. Staroverov, R. Kobayashi, J. Normand, K. Raghavachari, A. Rendell, J. C. Burant, S. S. Iyengar, J. Tomasi, M. Cossi, N. Rega, J. M. Millam, M. Klene, J. E. Knox, J. B. Cross, V. Bakken, C. Adamo, J. Jaramillo, R. Gomperts, R. E. Stratmann, O. Yazyev, A. J. Austin, R. Cammi, C. Pomelli, J. W. Ochterski, R. L. Martin, K. Morokuma, V. G. Zakrzewski, G. A. Voth, P. Salvador, J. J. Dannenberg, S. Dapprich, A. D. Daniels, O. Farkas, J. B. Foresman, J. V. Ortiz, J. Cioslowski and D. J. Fox, *Gaussian 09, Revision A.02*, Gaussian, Inc., Wallingford, CT, 2009
